# Frost-fighter, SVALKA-PRC2: winter, bring it on!

**DOI:** 10.1093/plphys/kiae057

**Published:** 2024-02-03

**Authors:** Divya Mishra

**Affiliations:** Assistant Features Editor, Plant Physiology, American Society of Plant Biologists; National Institute of Plant Genome Research, New Delhi 110067, India

How does the plant survive in nature's extremes? Plants possess different adaptive mechanisms, such as developing tolerance to low temperatures to thrive in harsh freezing conditions, a process known as cold acclimation. One of the major players in cold acclimation is C-REPEAT BINDING FACTORS (CBFs), a family of three tandemly duplicated CBF paralogs that activate cold-inducible genes (Novillo et al. 2007). The CBF family is induced during low temperatures, and its expression reaches the maximum in the initial phase of low temperatures, further decreasing in the later stages of low temperatures (Medina et al. 1999). Although the role of CBFs in cold induction has been extensively investigated, the underlying mechanisms of decline in CBF expression in later stages of low-temperature conditions remain elusive.

In this issue of *Plant Physiology*, Gómez-Martínez et al. (2023) demonstrated the underlying mechanism of how CBF expression declines during cold acclimation. Previous investigations showed that CURLY LEAF (CLF), a subunit of the Polycomb Repressive Complex 2 (PRC2), which promotes the formation of the H3K27me3 repressive mark, is associated with the genomic region of CBFs (Sequeira-Mendes et al. 2014). Therefore, the authors hypothesized that CLF decreases CBF expression via the deposition of repressive marks during cold acclimation. To address this, they showed a significant induction of *CBF1* and *CBF3* during the later phase of cold exposure in the *clf* mutants compared to the wild type (WT). Additionally, they found that after long cold exposure, the chromatin region of *CBF3* possesses a high abundance of repressive marks and is substantially associated with CLF. These findings indicate CLF is associated with the chromatin region of *CBF3* after long cold exposure.

Next, the authors deciphered the underlying mechanism of association between CLF and *CBF3* during cold acclimation. Long noncoding RNAs (lncRNAs) have been shown to contribute to PRC2 functionality (Margueron and Reinberg 2011). Kindgren et al. (2018) found one such lncRNA, *SVALKA*, transcribed from the antisense strand between *CBF3* and *CBF1* (Kindgren et al. 2018). The transcription of *SVALKA* isoforms exhibited maximum expression in the later stages of cold exposure, indicating its potential role in the decreased expression of *CBF3* during cold acclimation via recruitment of CLF onto *CBF3*. Therefore, the authors performed RNA immunoprecipitation and showed that *SVALKA* physically interacts with CLF after long cold exposure. Further, the authors performed chromatin immunoprecipitation to understand whether the *SVALKA*-CLF interaction is crucial for CLF recruitment to the *CBF3* coding region. They observed that a lower level of the repressive mark H3K27me3 was deposited on the *CBF3* coding region, and significantly fewer CLFs were associated with *CBF3* in *svalka* mutants compared to WT after long cold exposure.

These findings demonstrated that *SVALKA* is essential for the recruitment of CLF on *CBF3* and hence promotes the deposition of repressive mark H3K27me3, which decreases the *CBF3* expression during cold acclimation (Fig.). Collectively, this study unraveled the interaction of *SVALKA*-PRC2, which precisely modulates *CBF3* downregulation during cold acclimation and provides mechanistic insight into how plants adapt to temperature extremes.

**Figure.**
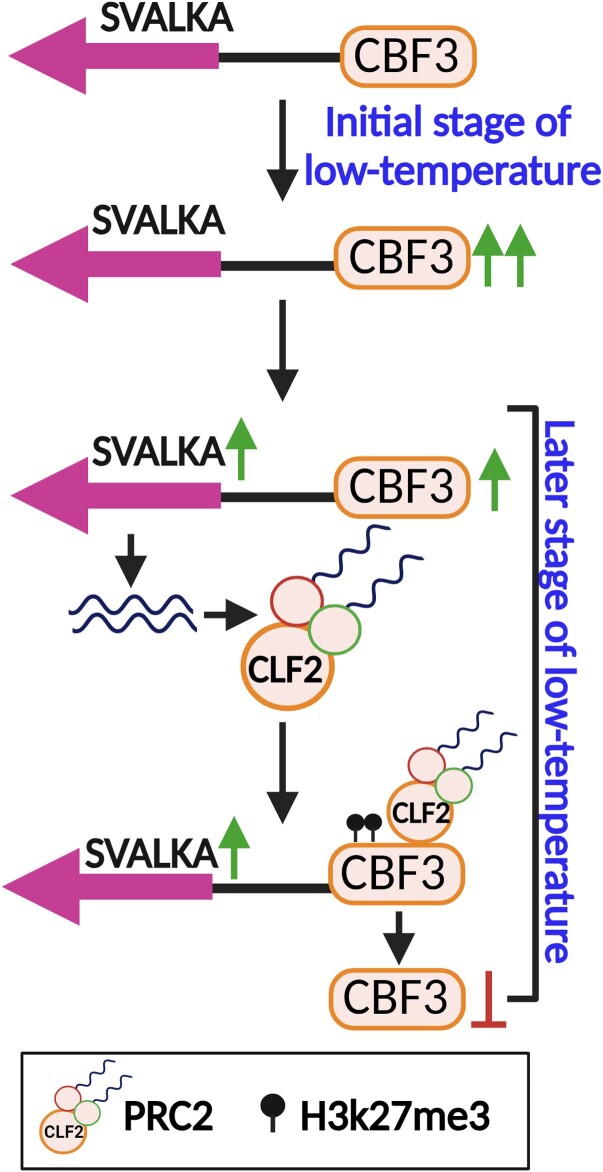
SVALKA-PRC2 interaction controls the recruitment of CLF. Under low temperatures, CBF expression level rises at the early stages and SVALKA expression rises in the later stages of cold exposure. *SVALKA* recruits the PRC2 subunit to the *CBF3* coding region and promotes the deposition of H3K27me3 that represses the *CBF3*. The figure is adapted and modified from Gómez-Martínez et al. 2023 and created by Biorender.com.

## Dive Curated Terms

The following phenotypic, genotypic, and functional terms are of significance to the work described in this paper:

clf Gramene: AT2G23380
clf Araport: AT2G23380

CBF3 Gramene: At4g25480
CBF3 Araport: At4g25480

CBF1 Gramene: Solyc03g026280
CBF1 Araport: Solyc03g026280
